# 

**Published:** 1883-11

**Authors:** 


### CONTENTS.

### ORIGINAL.

Idiopathic Tetanus. By W. A. Cook, M.D. .... 33
A Convenient Nozzle-Cap for the Urethral Syringe. By Jno. Thad. Johnson, M.D., .... 35

### EXTRACTS.

Impotence and Nocturnal Emissions Treated by Galvanism .... 36
The Therapeutics of Bluff .... 41
The Treatment of Acute Rheumatism .... 42
Convallaria Majalis in Heart Disease .... 45
Suburban Homes .... 46
What Surgery Can Do .... 48
The Use and Abuse of Bathing .... 49
Byron's Death .... 50
Heavy Damages Against a Chemist .... 50
Eczema of the Scalp in Infants .... 51
Hot Water in Epistaxis .... 51

### EDITORIAL.

Practical Chemistry for Students and Physicians—Chapter Fifth .... 52
Exercise Questions on Chapter Fifth .... 54
Dr. James Marion Sims .... 55
Patent Medicines in Atlanta .... 59
A Premium of 100,000 Dollars .... 60
Poisonous Oysters .... 61
The Medical Age .... 62
Rare Chance for a Doctor .... 62
BOOKS .... 62

